# Ascomycin enhances the antitumor activity of osimertinib in EGFR-TKI-resistant non-small cell lung cancer

**DOI:** 10.3389/fonc.2026.1867321

**Published:** 2026-08-19

**Authors:** Hongbing Zhang, Chen Chen, Di Wu, Weiting Li, Hao Gong, Chao Liu, Hongfeng Wu, Quan Zhang, Hongyu Liu, Jun Chen, Yongwen Li

**Affiliations:** 1 Tianjin Key Laboratory of Lung Cancer Metastasis and Tumor Microenvironment, Tianjin Lung Cancer Institute, Tianjin Medical University General Hospital, Tianjin, China; 2 Department of Lung Cancer Surgery, Tianjin Medical University General Hospital, Tianjin, China

**Keywords:** ascomycin, epidermal growth factor receptor-tyrosine kinase inhibitor (EGFR-TKI) resistance, interleukin-6/signal transducer and activator of transcription 3 (IL-6/STAT3) signaling, non-small cell lung cancer, osimertinib

## Abstract

**Background:**

Resistance to epidermal growth factor receptor tyrosine kinase inhibitors (EGFR-TKIs) remains a major challenge in non-small cell lung cancer (NSCLC) treatment. Although ascomycin (FK520) has demonstrated some antitumor activity, its potential in overcoming osimertinib resistance has not been fully characterized.

**Methods:**

Cell viability and proliferation were assessed using the Cell Counting Kit 8 (CCK8) and colony formation assays, respectively, following treatment with ascomycin, osimertinib, or interleukin (IL)-6 for 48 h. Flow cytometry was employed to characterize cell cycle distribution and apoptotic rates. To identify key regulatory pathways, transcriptomic profiling was performed via RNA sequencing. The expression of relevant molecular targets was quantified using western blotting, and quantitative PCR (qPCR). Finally, the chemosensitizing effect of ascomycin was validated in vivousing established animal models.

**Results:**

Ascomycin were observed to significantly enhanced the antiproliferative effects of osimertinib in EGFR-TKI-resistant lung cancer cells. Further analysis indicated that such effects may be mediated, at least in part, by modulating the interleukin-6/signal transducer and activator of transcription 3 (IL-6/STAT3) signaling pathway and promoting tumor cell apoptosis. Consistent with *in vitro* results, combined ascomycin and osimertinib treatment generated greater antitumor efficacy than either agent alone in osimertinib-resistant NSCLC xenograft models.

**Conclusions:**

Ascomycin may be an effective adjuvant in enhancing osimertinib therapeutic activity and overcoming acquired EGFR-TKI resistance, thereby providing a promising combination strategy for treating resistant NSCLC.

## Introduction

Lung cancer remains the most commonly diagnosed malignancy and the leading cause of cancer-related mortality worldwide. According to GLOBOCAN 2022, approximately 2.5 million new cases and 1.8 million deaths were globally attributed to lung cancer in 2022, underscoring a substantial public health burden (1). Non-small cell lung cancer (NSCLC) accounts for approximately 85%–90% of all lung cancer cases. Despite significant therapeutic advances, 5-year survival rates for patients with advanced NSCLC remains low, ranging from 10% to 15% depending on disease stage and geographical region. Critically, the discovery of activating mutations in the epidermal growth factor receptor (*EGFR*) gene has transformed the NSCLC treatment landscape. EGFR tyrosine kinase inhibitors (EGFR-TKIs) have become the standard first-line therapy for patients harboring EGFR-activating mutations and provide substantial clinical benefits when compared with conventional chemotherapy (2, 3). However, the efficacy of EGFR-TKIs, including the third-generation inhibitor osimertinib, remains limited by acquired resistance development. This multifactorial process commonly involves secondary EGFR mutations, and activated alternative bypass signaling pathways (4, 5). Increasing evidence now indicates signal transducer and activator of transcription 3 (STAT3) as a critical mediator of compensatory signaling that promotes tumor cell survival during EGFR-TKI treatment (6, 7). Nevertheless, the molecular mechanisms whereby STAT3 contributes to osimertinib resistance remain unclear, and the identification of agents that target this pathway is a key clinical objective.

Ascomycin (FK520) is a 23-membered macrocyclic polyketide isolated from *Streptomyces hygroscopicus* var. *ascomyceticus* and is widely used as a potent immunosuppressive agent (8, 9). Ascomycin is a potent calcineurin (CaN) phosphatase inhibitor that suppresses T-cell activation by inhibiting the dephosphorylation of nuclear factor of activated T cells (NFAT), thereby preventing the transcription of cytokine genes essential for immune responses. Clinically, the polyketide is efficacious in treating autoimmune and dermatological disorders and preventing allograft rejection following organ transplantation (10, 11). Recent studies report that ascomycin has more broader pharmacological activities, including antitumor effects, e.g., the polyketide inhibits melanoma cell growth and invasion by modulating Nuclear Factor of Activated T Cells 3 (NFAT3) signaling (12). However, it remains unclear whether ascomycin enhances EGFR-TKI efficacy or overcomes acquired resistance in NSCLC.

Given the urgent need for effective strategies that extend EGFR-TKI responses, ascomycin were evaluated as a sensitizing agent in osimertinib-resistant NSCLC models. Particular attention was directed toward the interleukin (IL)-6/STAT3 signaling pathway as a potential, therapeutic response mediator. The present findings provide mechanistic insights into how ascomycin reverses acquired resistance in NSCLC and establish a rationale for the development of ascomycin-based combination therapies for EGFR-TKI-resistant NSCLC.

## Materials and methods

### Cell lines and reagents

The human NSCLC cell line H1975 was obtained from the American Type Culture Collection (Manassas, VA, USA). Cells were authenticated by short tandem repeat profiling and confirmed as free of mycoplasma contamination before use. The osimertinib-resistant cell line H1975-AR was established in-house using a stepwise dose-escalation protocol. Ascomycin (purity >98%) was generously provided by Prof. Jianping Wen (Tianjin University) (13, 14). Osimertinib was purchased from Selleck Chemicals (Houston, TX, USA; Cat. No. S7297). Roswell Park Memorial Institute 1640 medium and fetal bovine serum were obtained from Gibco (Carlsbad, CA, USA). Recombinant human IL-6 was purchased from Sino Biological (Beijing, China). Primary antibodies against p53, Bax, Ki-67, caspase-3, and β-actin were purchased from Proteintech Group (Wuhan, China). Antibodies against Akt, STAT3, phospho-JAK1, phospho-JAK2, phospho-STAT3 (Tyr705), and phospho-Akt (Ser473) were obtained from Cell Signaling Technology (Danvers, MA, USA). Horseradish peroxidase (HRP)-conjugated goat anti-rabbit and anti-mouse IgG secondary antibodies were purchased from ABclonal (Wuhan, China). Cell Counting Kit-8 (CCK-8) was obtained from ApexBio (Boston, MA, USA).

### Cell proliferation and viability assays

TKI-sensitive H1975 and TKI-resistant H1975-AR cells were seeded in 96-well plates at 5 × 10^3^ cells/well. After incubation for 24 h to allow attachment, cells were treated with indicated concentrations of test compounds or a vehicle control (dimethyl sulfoxide (DMSO)) for 48 h. Subsequently, 10 μL of CCK-8 solution was added to wells, followed by incubation at 37 °C for 2 h. Absorbance was measured at 450 nm using an ELx800 microplate reader (BioTek, Winooski, VT, USA). The half-maximal inhibitory concentration (IC50) was determined by nonlinear regression analysis in GraphPad Prism 9.5 (GraphPad Software, San Diego, CA, USA). The dose-effect curve and combination index (CI) was calculated using the CalcuSyn software (Biosoft, Ferguson, MO, USA). Data were confirmed by performing at least three independent experiments.

### Colony formation assays

Assays were performed as previously described (15). Briefly, approximately 500 cells were seeded into wells in 6-well plates and allowed to attach for 24 h. Cells were then treated with indicated drugs or vehicle control for 24 h. The drug-containing medium was then replaced with fresh complete medium, which was replenished every 3 days. After 14 days, colonies were fixed in 4% paraformaldehyde for 20 min and stained in 0.5% crystal violet for 15 min. Colonies with more than 50 cells were counted under a light microscope (IX73,Olympus, Tokyo, Japan).

### Cell cycle and apoptosis analyses

H1975-AR cells were seeded into 6-well plates and treated as indicated. For apoptosis analyses, cells were collected after 48 h of treatment, washed twice in cold phosphate-buffered saline (PBS), and stained using an Annexin V-Fluorescein isothiocyanate (FITC)/propidium iodide (PI) Apoptosis Detection Kit (BD Biosciences, San Jose, CA, USA). For cell cycle analyses, cells were fixed overnight in 75% cold ethanol at -20 °C and stained in PI solution plus RNase (BD Biosciences, San Jose, CA, USA). Data were acquired using a NovoCyte flow cytometer (Agilent Technologies, Santa Clara, CA, USA) and analyzed in NovoExpress software. All experiments were performed in triplicate.

### siRNA transfection

STAT3 knockdown was carried out as previously described (15). Briefly, cells were transfected with STAT3-specific siRNA or a scrambled negative control siRNA (Ribobio, Guangzhou, China) using Lipofectamine 2000 (Invitrogen, Carlsbad, USA) according to the manufacturer’s guidelines. The sequences of the STAT3-targeting siRNA were as follows: sense strand, 5’-CCGUGGAACCAUACACAAA-3’; antisense strand, 5’-GGCACCUUGGUAUGUGUUU-3’.

### Western blotting

Cells were lysed in RIPA buffer (Beyotime, Shanghai, China) containing protease and phosphatase inhibitor cocktails. Protein concentrations were determined using a Bicinchoninic Acid Assay Protein Assay Kit (Pierce, Rockford, IL, USA). Equal protein quantities (30 μg/lane) were separated by 10% or 12% sodium dodecyl sulfate-polyacrylamide gel electrophoresis, transferred to polyvinylidene difluoride membranes (Millipore, Billerica, MA, USA), blocked in 5% nonfat milk for 1 h at room temperature, and incubated overnight at 4 °C with indicated primary antibodies diluted in TBST containing 1% bovine serum albumin. After three washes in TBST, membranes were incubated with appropriate HRP-conjugated secondary antibodies (1:5,000; ABclonal, Wuhan, China) for 1 h at room temperature. Protein bands were visualized using an enhanced chemiluminescence detection kit (Millipore, MA, USA) and imaged using a gel documentation system (Syngene, Cambridge, UK). Protein expression levels were quantified by densitometric analysis and normalized to β-actin levels. Relative expression levels were calculated against a control group (assigned 1.0).

### Real-time quantitative polymerase chain reaction

Total RNA was extracted from cells using a SparkEasy Tissue/Cell RNA Kit (SparkJade, Shandong, China), after which 1 μg of total RNA was reverse-transcribed to cDNA using a Reverse Transcription System (Takara, Dalian, China). RT-qPCR was performed using ChamQ Universal SYBR qPCR Master Mix (Vazyme, Nanjing, China) on an ABI 7500 Real-Time PCR System. Relative mRNA expression levels were calculated using the 2^−ΔΔCt^ method, with β-actin the internal reference gene. Primers were synthesized by BGI (Beijing, China) and are listed (**Table 1**).

**Table 1:** Primers used for qPCR.

Primers		Sequence
IL6	Forward	5’-GCCACTCACCTCTTCAGAACG-3’
	Reverse	5’-TGCCTCTTTGCTGCTTTCA-3’
IL6R	Forward	5’-GGTCAAGGACCTCCAGCATC-3’
	Reverse	5’-CTTGCCCGAACTCCTCCTG-3
gp130	Forward	5’-ACAGCATCCAGTGTCACCTTT-3’
	Reverse	5’-TCTGTTCAAGCTGTCCGAATGT-3’
JAK2	Forward	5’-CCAGATGGAAACTGTTCGCTCAG-3’
	Reverse	5’-GAGGTTGGTACATCAGAAACACC-3’
STAT3	Forward	5’-TCCATCAGCTCTACAGTGACAGC-3’
	Reverse	5’-TCCCAGGAGATTATGAAACACC-3’
β-actin	Forward	5’-ATAGCACAGCCTGGATAGCAACGTAC-3’
	Reverse	5’-CACCTTCTACAATGAGCTGCGTGTG-3’

### Animal studies

Four-week-old female BALB/c nude mice (11–15 g) came from Beijing Vital River Laboratory Animal Technology Co., Ltd. (Beijing, China) and were maintained under controlled environmental conditions (Experimental Animal Use License No. SYXK [Tianjin] 2016-0012). To establish xenograft models, 5×10^6^ H1975-AR cells were subcutaneously injected into the right flank of each mouse. Tumor volume was calculated using the formula: volume = (length × width²)/2. When tumors reached approximately 100 mm³, mice were randomly assigned to four groups (n = 6/group) and treated by oral gavage every 2 days for 28 days as follows: (1) vehicle control; (2) ascomycin (10 mg/kg); (3) osimertinib (2.5 mg/kg); and (4) ascomycin (10 mg/kg) plus osimertinib (2.5 mg/kg). Mice were humanely euthanized when the tumor volume reached 1,500 mm³ or at study end (day 28). Euthanasia was performed by intraperitoneal tribromoethanol administration (250 mg/kg; Sigma-Aldrich, St. Louis, MO, USA), followed by cervical dislocation. Tumors were excised, weighed, and underwent paraffin embedding. Animal procedures were approved by the Medical Ethics Committee of Tianjin Medical University General Hospital (Approval No. IRB2021-DWFL-087).

### Immunohistochemistry

IHC staining was performed as previously described (15). Briefly, paraffin-embedded tissue sections (4 μm) were deparaffinized and rehydrated. Antigen retrieval was performed in citrate buffer (pH 6.0) in a pressure cooker for 3 min. Endogenous peroxidase activity was blocked with 3% hydrogen peroxide. Sections were incubated overnight at 4 °C with the following primary antibodies: rabbit anti-p53 (1:500; Proteintech Group, Wuhan, China), rabbit anti-Ki-67 (1:4,000; Proteintech Group), and rabbit anti-phospho-STAT3 (Tyr705; 1:100; Cell Signaling Technology, Danvers, MA, USA). After washing, sections were incubated with appropriate HRP-conjugated secondary antibodies. Immunoreactivity was visualized using a 3, 3’-diaminobenzidine detection kit (Beyotime, Shanghai, China), followed by hematoxylin counterstaining. The percentage of positively stained cells was scored as follows: 1 (0%–25%); 2 (26%–50%); 3 (51%–75%); and 4 (>75%). Staining intensity was graded as 0 (negative), 1 (weak), 2 (moderate), or 3 (strong). The final IHC score was calculated by multiplying the percentage score by the intensity score, yielding a total score in the 0–12 range.

### RNA sequencing

H1975-AR cells were treated with indicated drugs or a vehicle control for 24 h. Total RNA was extracted using a SparkEasy Tissue/Cell RNA Kit (SparkJade, Shandong, China) and samples submitted to BGI (Wuhan, China) for library preparation and sequencing. Transcriptome sequencing was performed on the BGISEQ-500 platform. Differential expression genes (DEGs), Kyoto Encyclopedia of Genes and Genomes (KEGG) pathway enrichment, and Gene Ontology (GO) enrichment analyses were conducted using the MyBGI 3.0 platform (BGI, Shenzhen, https://mybgi.bgi.com/tech/).

### Statistical analysis

Data were presented as the mean ± standard deviation (SD) from at least three independent experiments. Statistical analyses were performed in SPSS version 21.0 (SPSS Inc., Chicago, IL, USA) and GraphPad Prism 9.5 (GraphPad Software, San Diego, CA, USA). Differences between two groups were assessed using two-tailed Student’s t-tests, whereas comparisons among multiple groups were performed using one-way analysis of variance (ANOVA) followed by Tukey’s *post hoc* tests. A two-sided P value < 0.05 was considered statistically significant.

## Results

### Ascomycin enhances osimertinib anti-proliferative activity in TKI-resistant lung cancer cells

**Figure 1:**
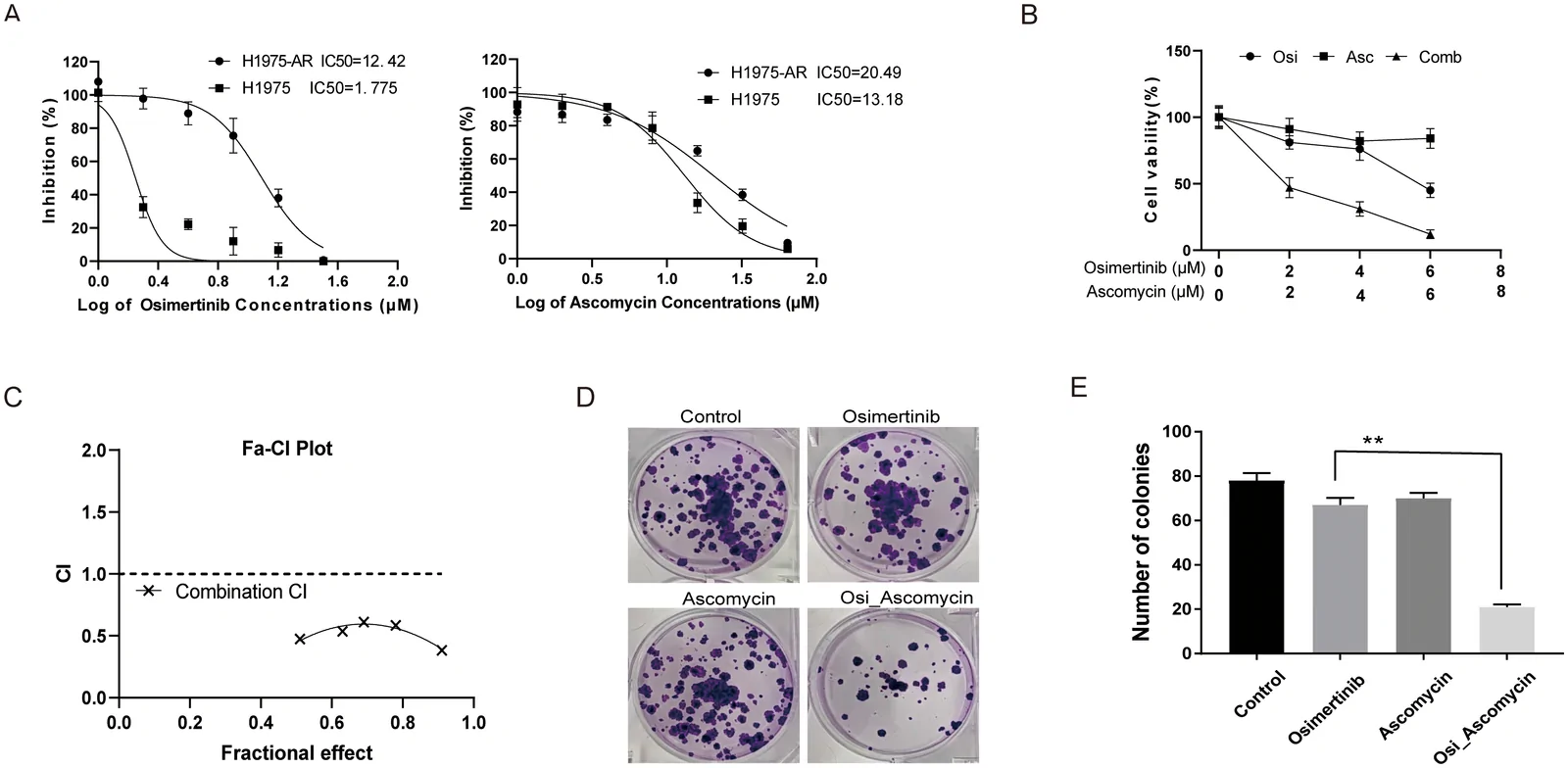
Ascomycin enhances osimertinib antiproliferative activity in TKI-resistant lung cancer cells. **(A)** Cell proliferation of parental (H1975) and resistant (H1975-AR) cell lines after 48-hour treatment with designated doses of osimertinib or ascomycin was evaluated using the CCK8 assay. The half-maximal inhibitory concentration (IC50) for each cell line is indicated. **(B)** H1975-AR cells treated with Osimertinib (0-6 μM) and ascomycin (0-6 μM) for either alone or in combination for 48 hours were detected using CCK8 assay. Cells treated with DMSO served as the control group. Osi:osimertinib, Asc:ascomycin, Comb:.combination of the two drugs. **(C)** The combination of osimertinib and ascomycin at combined concentrations of 2 μM + 2 μM, 3 μM + 3 μM, 4 μM + 4 μM, 5 μM + 5 μM and 6 μM + 6 μM induced significant synergistic cytotoxicity in H1975-AR cells, as analyzed by Calcusyn Software [Combination index (CI) <1.0]. A CI value <1.0 indicates a synergistic interaction. **(D, E)** H1975-AR cells were treated with vehicle (DMSO), 4μM osimertinib, 4μM ascomycin, or the combination of both drugs for 15 days, followed by a colony formation assay analysis. Quantitative data are presented as the mean ± SD from three independent experiments. *p < 0.05, **p < 0.01, one-way ANOVA.

Parental H1975 and osimertinib-resistant derivative H1975-AR cell sensitivity to osimertinib and ascomycin was first evaluated via CCK-8 assays. Specifically, the IC50 of osimertinib was 1.775 in parental H1975 cells and 12.42 in H1975-AR resistant cells, indicating that H1975-AR cells exhibited significantly enhanced osimertinib resistance, with an approximate 7.0-fold increase in drug resistance compared with the parental cell line. Meanwhile, the IC50 values of ascomycin against H1975 and H1975-AR cells were 13.18 and 20.49, respectively (**Figure 1A**). To determine whether ascomycin enhanced osimertinib antitumor activity in TKI-resistant cells, H1975-AR cells with various concentrations of osimertinib alone, ascomycin alone, or the two drugs combination for 48 h. Combined treatment with osimertinib and ascomycin exerted stronger anti-proliferative effects on H1975-AR cells than either monotherapy (**Figure 1B**). The Chou-Talalay method was utilized to determine combination index values and assess the combined antiproliferative activity. All detected CI values were < 1, demonstrating synergism between ascomycin and osimertinib (**Figure 1C**; **Table 2**). To further evaluate the long-term treatment effects on cell proliferative capacity, colony formation assays were performed. Consistent with CCK-8 assays, osimertinib and ascomycin monotherapy barely inhibited colony formation of H1975-AR cells, reaching 90.3% and 85.1% of control levels, respectively. Combined treatment reduced clonogenic activity to 27.3% of the control, which was merely 0.29-fold of the osimertinib group, corresponding to a 70.77% decrease. This represented a significant decrease when compared with osimertinib or ascomycin monotherapy. Taken together, ascomycin significantly enhanced osimertinib antiproliferative activity in EGFR-TKI-resistant NSCLC cells.

**Table 2:** Synergistic analysis of osimertinib and ascomycin combination in H1975-AR cells.

Drug treatment	IC50 (μM)	CI value	DRI (osimertinib)	DRI (ascomycin)
Osimertinib alone	12.42	N/A	N/A	N/A
Ascomycin alone	20.49	N/A	N/A	N/A
Osi + Asc: 2μM+2μM	N/A	0.47397	3.19342	6.21795
Osi + Asc: 3μM+3μM	N/A	0.53526	2.83188	5.49022
Osi + Asc: 4μM+4μM	N/A	0.61152	2.48073	4.79813
Osi + Asc: 5μM+5μM	N/A	0.58443	2.59936	5.00708
Osi + Asc: 6μM+6μM	N/A	0.38323	3.97652	7.58972

### Ascomycin enhances osimertinib antitumor activity by promoting apoptosis in TKI-resistant lung cancer cells

**Figure 2:**
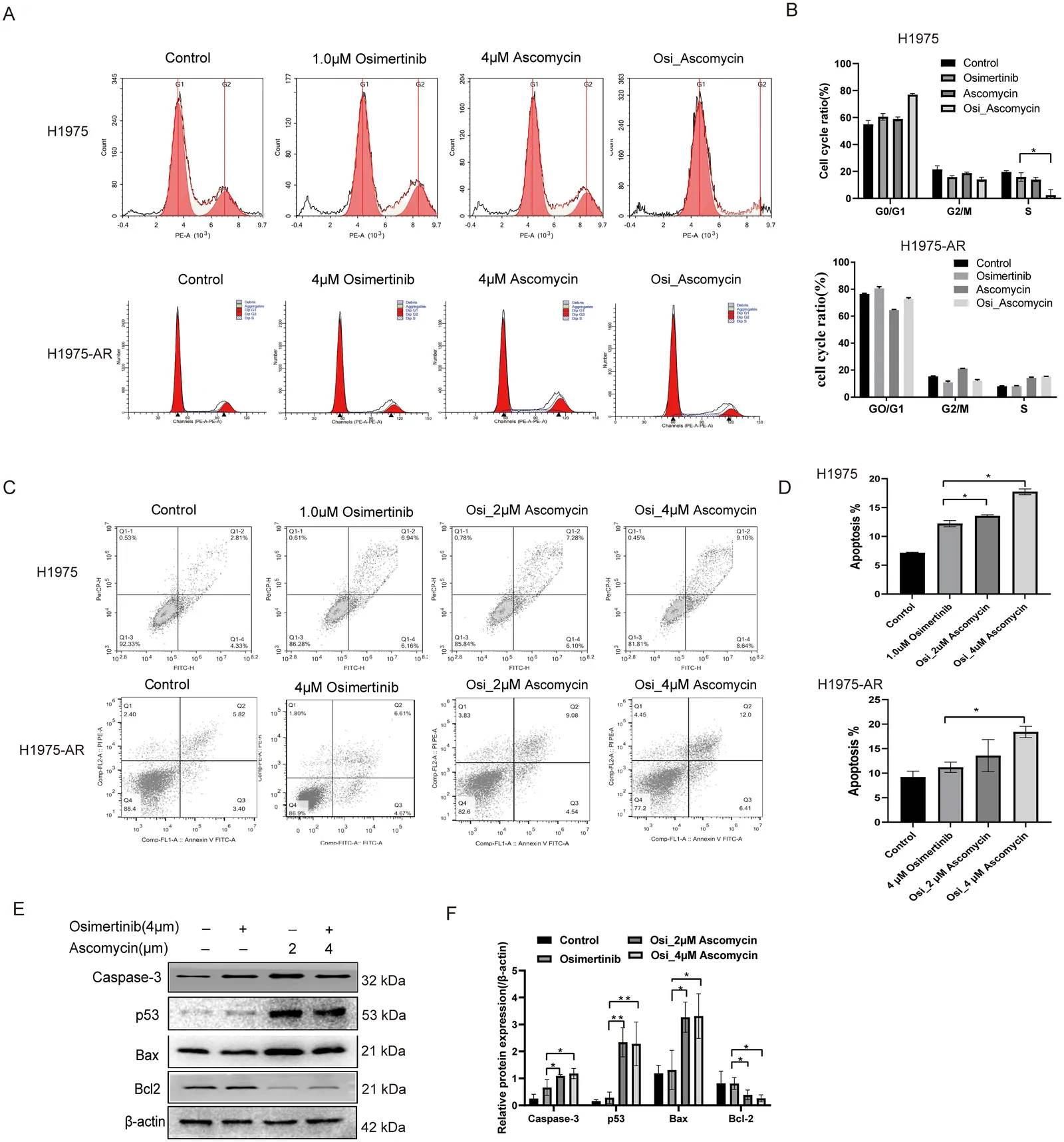
Ascomycin overcomes osimertinib resistance by inducing apoptosis. **(A, B)** H1975 and H1975-AR cells were treated with vehicle (DMSO), 1 μM or 4 μM osimertinib, 4 μM ascomycin, or the combination for 48 h. Cell cycle distribution was analyzed by flow cytometry, with corresponding quantitative results shown in the right panels. **(C, D)** Apoptosis was assessed by Annexin V-FITC/PI staining and flow cytometry after 48 h. Quantitative results are shown in right panels. **(E)** p53, Bax, Bcl-2 and cleaved caspase-3 expression was determined by western blotting following 48 h of treatment. Quantitative data are presented as the mean ± SD (n = 3).

To investigate the mechanism underlying the growth-inhibitory effects of the combination treatment, cell cycle distribution and apoptosis processes were examined in H1975 and H1975-AR cells. Given that the IC50 of osimertinib in parental H1975 cells was much lower than that in resistant H1975-AR cells, H1975 cells were treated with 1.0 μM osimertinib, while H1975-AR cells received 4 μM ascomycin and 4 μM osimertinib for intervention. Flow cytometric analysis of PI-stained cells showed that neither ascomycin nor osimertinib, alone or in combination, induced significant cell cycle distribution alterations. Notably, in H1975 cells, the proportion of cells in S phase was markedly reduced in the combination group compared with osimertinib alone group (**Figures 2A, B**), suggesting that growth suppression was not mediated via cell cycle arrest in H1975-AR cells. Apoptosis was then assessed using Annexin V-FITC/PI double staining. In H1975-AR cells, osimertinib alone barely induced apoptosis: the apoptotic rate was 11.2%, compared with 8.3% in the control group. In contrast, combined ascomycin and osimertinib treatment markedly increased apoptotic cell proportions (**Figures 2C, D**). Specifically, 18.4% of cells underwent apoptosis following 4 μM ascomycin plus 4μM osimertinib combination treatment, representing a 1.6-fold increase relative to the osimertinib alone group. For parental H1975 cells with lower osimertinib IC50, 1μM osimertinib was applied. Both single osimertinib and cotreatment effectively triggered obvious apoptosis in H1975 cells. To further validate these findings, apoptosis-related protein expression was examined by western blotting. Consistent with flow cytometric results, osimertinib monotherapy exerted mild effects on apoptosis-related proteins versus the control. By contrast, ascomycin and osimertinib co-treatment substantially increased caspase-3 cleavage and elevated p53 (7.1-fold increase) and Bax (1.5-fold increase) expression levels when compared with osimertinib treatment alone, while downregulating the expression of Bcl-2 (0.68-fold decrease) (**Figure 2E**). Collectively, ascomycin appeared to enhance osimertinib antitumor activity in H1975-AR cells primarily via induced apoptosis rather than through modulated cell cycle progression.

### The IL-6/STAT3 axis is involved in osimertinib-resistant cell responses to ascomycin

**Figure 3:**
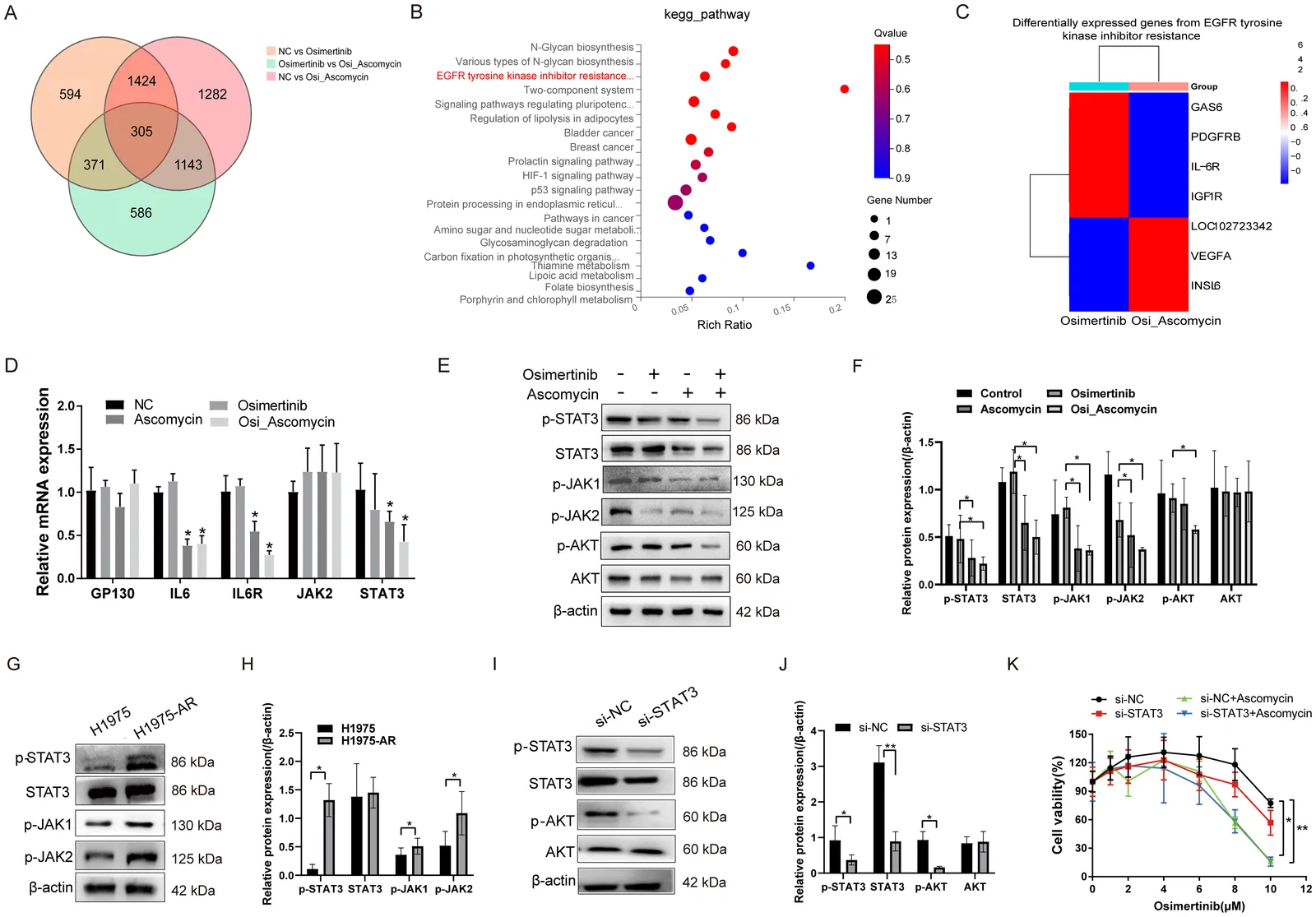
Ascomycin suppresses IL-6/JAK/STAT3 signaling in osimertinib-resistant cells. **(A)** Venn diagram showing overlapping candidate genes identified by RNA-Seq of H1975-AR cells treated with osimertinib, ascomycin, or the combination. **(B)** KEGG pathway enrichment analysis of candidate differentially expressed genes. **(C)** Heatmap showing gene expression profiles associated with EGFR-TKI resistance, including *GAS6, PDGFRB, IL-6R, IGF1R, LOC102723342, VEGFA*, and *INSL6*. **(D)** Relative mRNA expression levels of indicated genes by RT-qPCR (n = 3). **(E)** Western blotting showing total and phosphorylated STAT3, JAK1, JAK2, and AKT levels following indicated treatments. **(F)** Quantification of protein expression normalized to β-actin levels. **(G)** STAT3, p-STAT3, JAK2, and p-JAK2 protein expression levels in H1975 and H1975-AR cells by western blotting. **(H)** Quantification of protein expression normalized to β-actin levels. **(I)** H1975-AR cells were transfected with control or STAT3 siRNA for 48 h. STAT3, p-STAT3, p-JAK1, and p-JAK2 expression levels were analyzed by western blotting. **(J)** Quantification of protein expression normalized to β-actin levels. **(K)** H1975-AR cells were treated with vehicle control, STAT3 siRNA, 4 μM ascomycin, or STAT3 siRNA plus ascomycin for 48 h. Cell viability was assessed using CCK-8 assays. Quantitative data are presented as the mean ± SD (n = 3). *p < 0.05, **p < 0.01, one-way ANOVA.

To elucidate the molecular mechanisms whereby ascomycin enhanced osimertinib antiproliferative effects, RNA-seq was performed in H1975-AR cells treated with vehicle control, ascomycin, osimertinib, or the combination. Differential expression analysis identified 4,154 and 2,694 differentially expressed genes (DEGs) in ascomycin- and osimertinib-treated cells, respectively, when compared with the vehicle control group. A comparison between the combination treatment group and the osimertinib monotherapy group identified 2,405 DEGs. To identify genes uniquely regulated by the combination, Venn diagram analysis was performed, revealing 586 unique DEGs (**Figure 3A**). KEGG pathway enrichment analysis demonstrated that these 586 genes were significantly enriched across several pathways, with the “EGFR-TKI resistance” pathway among the most significantly enriched (**Figure 3B**). This pathway was therefore selected for further mechanistic investigation. Among the DEGs in this pathway, seven candidate genes associated with EGFR-TKI resistance were identified (**Figure 3C**; **Table 3**), including *PDGFRB* (platelet-derived growth factor receptor beta), *LOC102723342*, *INSL6* (insulin-like peptide 6), *VEGFA* (vascular endothelial growth factor A), *GAS6* (growth arrest-specific protein 6), *IGF1R* (insulin-like growth factor 1 receptor), and *IL-6R* (interleukin-6 receptor).

**Table 3:** The main different genes list of EGFR tyrosine kinase inhibitor resistance pathway.

Gene symbol	Osimertinib expression	Osi_ascomycin expression	log2	P value
PDGFRB	5.69	2.44	-1.22155	P<0.001
LOC102723342	0.8	1.81	1.17792	P<0.001
INSL6	0.19	0.42	1.14439	P<0.001
VEGFA	14.16	52.08	1.87891	P<0.001
GAS6	2.1	0.73	-1.52442	P<0.001
IGF1R	5.99	2.86	-1.06654	P<0.001
IL-6R	5.2	2.27	-1.19582	P<0.001

PDGFRB is a member of the platelet-derived growth factor (PDGF) receptor family and activates multiple downstream signaling pathways upon ligand binding (16) Transcriptional PDGFRB de-repression has been reported to promote EGFR-TKI resistance in glioma cells (17). *LOC102723342* is a gene of unknown function that is specifically induced by HBoV infection in CuFi-8 cells (18). *INSL6* is a member of the insulin superfamily and is involved in reproductive and metabolic regulation (19). *VEGFA* is a prototypical VEGF family member (20) that regulates pathological angiogenesis via interactions with VEGFR1, VEGFR2, and VEGFR3. *GAS6*, together with its receptor AXL (AXL Receptor Tyrosine Kinase), is frequently overexpressed in multiple cancer types and contributes to EGFR-TKI resistance in lung cancer cells (21, 22). GAS6/AXL signaling is involved in cell proliferation, survival, migration, invasion, angiogenesis, and immune evasion. IGF1R, which binds IGF1 and IGF2, is activated by several growth factors, including basic fibroblast growth factor and PDGF (23). Activated IGF1R participates in the regulation of cell growth and differentiation. IL-6R is a cell-surface receptor that specifically binds IL-6, a cytokine involved in diverse immune responses, with important roles in immune regulation (24). Given that ascomycin is a potent calcineurin (CaN) phosphatase inhibitor with well-established immunosuppressive activity, focused was directed toward IL-6R. The IL-6/IL-6R signaling axis is a well-recognized mediator of oncogenic pathway reactivation and therapeutic resistance in NSCLC, making it a compelling candidate for further investigation.

To validate IL-6 signaling involvement, key pathway component mRNA expression was examined by RT-qPCR. Co-treatment with ascomycin and osimertinib markedly attenuated IL-6, IL-6R, and STAT3 expression in H1975-AR cells relative to control conditions. Furthermore, the combined administration of ascomycin and osimertinib yielded a significant reduction in IL-6, IL-6R, and STAT3 levels compared to osimertinib monotherapy in these cells. Specifically, IL-6 mRNA levels decreased by 66% relative to the osimertinib monotherapy group, whereas IL-6R and STAT3 transcript levels were reduced by 59% and 44%, respectively (**Figure 3D**). Protein-level changes were also evaluated by western blotting (**Figures 3E, F**). Although ascomycin monotherapy partially reduced STAT3 phosphorylation, the combined ascomycin and osimertinib treatment effectively abolished both STAT3 and AKT phosphorylation (decreased by 54.1% and 36.2%, respectively), indicating a potent suppression of these oncogenic signaling pathways. Given that IL-6-mediated STAT3 activation is a critical therapeutic resistance driver in NSCLC (7, 25, 26), it was hypothesized that the IL-6/JAK/STAT3 signaling axis was possibly hyperactivated in H1975-AR cells. Immunoblotting confirmed that H1975-AR cells exhibited an 11-fold increase in phosphorylated STAT3 levels, along with increased JAK1 activity by 41.6% and JAK2 activity by 109.6% relative to parental H1975 cells (**Figures 3G, H**). To further assess a functional STAT3 role in TKI resistance, STAT3 expression was silenced using small interfering RNA (siRNA) (**Figures 3I, J**). Compared with the control group, STAT3 silencing alone exerted obvious inhibitory effects on H1975-AR cell viability. The combinatorial use of STAT3 knockdown and ascomycin further amplified this inhibitory activity (**Figure 3K**). Collectively, the IL-6/STAT3 signaling axis appeared to be aberrantly activated in osimertinib-resistant NSCLC cells and possibly contributed to increased cell sensitivity to osimertinib following ascomycin treatment.

### Ascomycin reverses IL-6-induced TKI resistance in TKI-sensitive lung cancer cells

**Figure 4:**
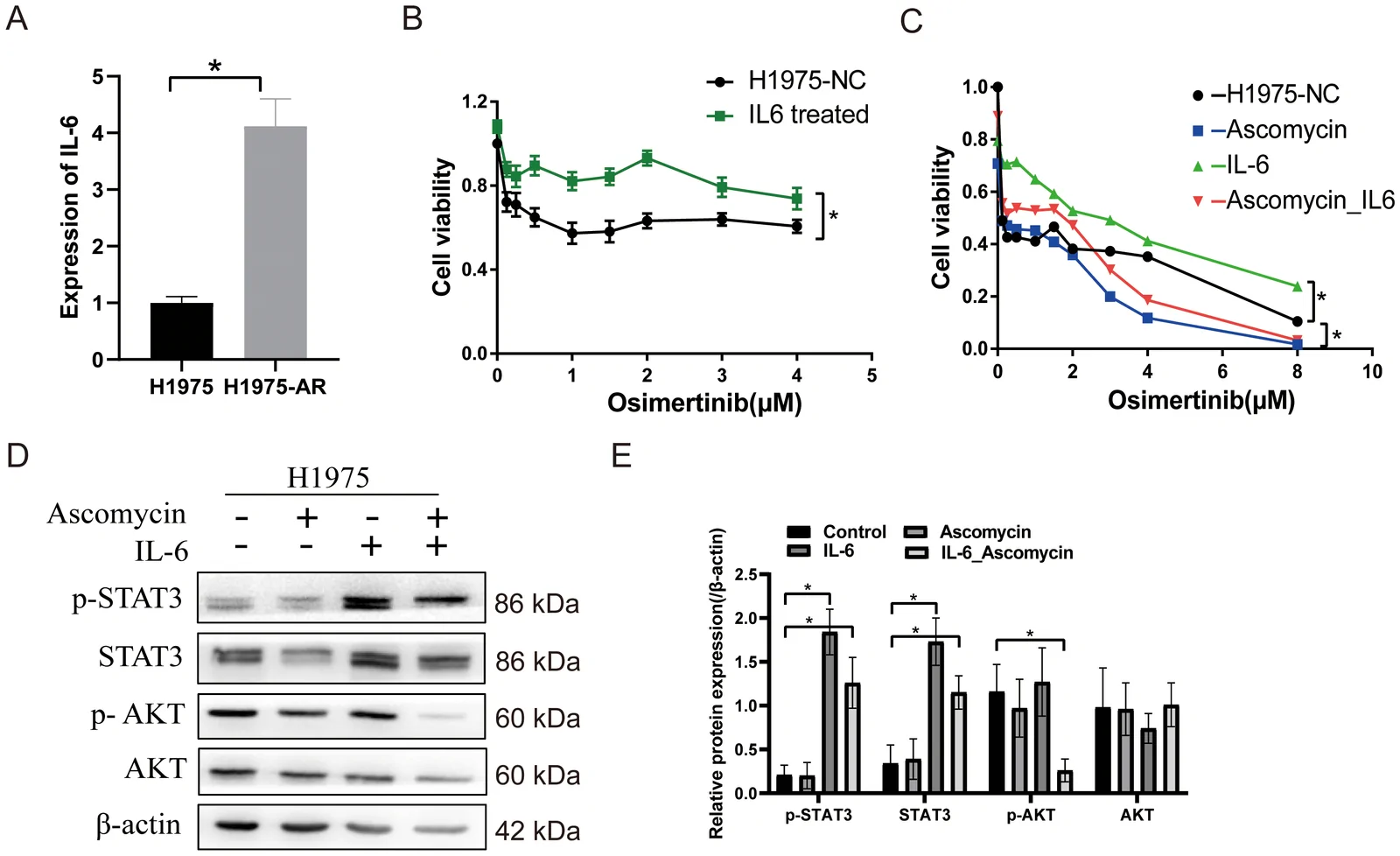
Ascomycin reverses IL-6-induced osimertinib resistance. **(A)** Relative IL-6 mRNA expression levels in parental H1975 and resistant H1975-AR cells by RT-qPCR (n = 3). **(B)** H1975 cells were treated with vehicle (DMSO) or IL-6 (10 ng/mL) for 48 h, with cell viability assessed using CCK-8 assays. **(C)** H1975 cells were treated with vehicle, IL-6 (10 ng/mL), 4 μM ascomycin, or IL-6 combined with ascomycin for 48 h. Cell viability was measured using CCK-8 assays. **(D)** STAT3, p-STAT3, AKT, and p-AKT expression was analyzed by western blotting following indicated treatments. **(E)** Quantification of protein expression normalized to β-actin. Data are presented as the mean ± SD from three independent experiments. *p < 0.05.

Given that IL-6 signaling is a well-established driver of acquired EGFR-TKI resistance (27), the modulation of IL-6-triggered osimertinib-resistant phenotypes by ascomycin was examined. RT-qPCR confirmed that IL-6 mRNA expression was significantly higher in H1975-AR cells when compared with parental H1975 cells, with expression levels approximately 4.1-fold greater in resistant cells (**Figure 4A**). To evaluate a functional role for IL-6, TKI-sensitive H1975 cells were treated with recombinant IL-6. As expected, IL-6 stimulation significantly reduced cell sensitivity to osimertinib (**Figure 4B**). Notably, ascomycin co-treatment effectively reversed IL-6-induced resistance and restored osimertinib sensitivity in H1975 cells (**Figure 4C**). These findings indicated that ascomycin effectively restored osimertinib sensitivity in IL-6-stimulated cells. To investigate the molecular basis underpinning these effects, the ascomycin impact on IL-6-mediated signaling was assessed by western blotting. Ascomycin monotherapy barely altered STAT3 and AKT activity. IL-6 stimulation induced a 7.76-fold increase in phosphorylated STAT3 levels, whereas co-treatment with ascomycin substantially attenuated this effect (**Figures 4D, E**). These results suggested that ascomycin antagonized IL-6-induced resistance by suppressing downstream STAT3 activation, thereby restoring lung cancer cell sensitivity to osimertinib. Collectively, these findings support IL-6/STAT3 signaling pathway involvement in regulating osimertinib responsiveness following ascomycin treatment.

### Ascomycin reverses osimertinib resistance by suppressing IL-6/JAK1/STAT3 signaling

**Figure 5:**
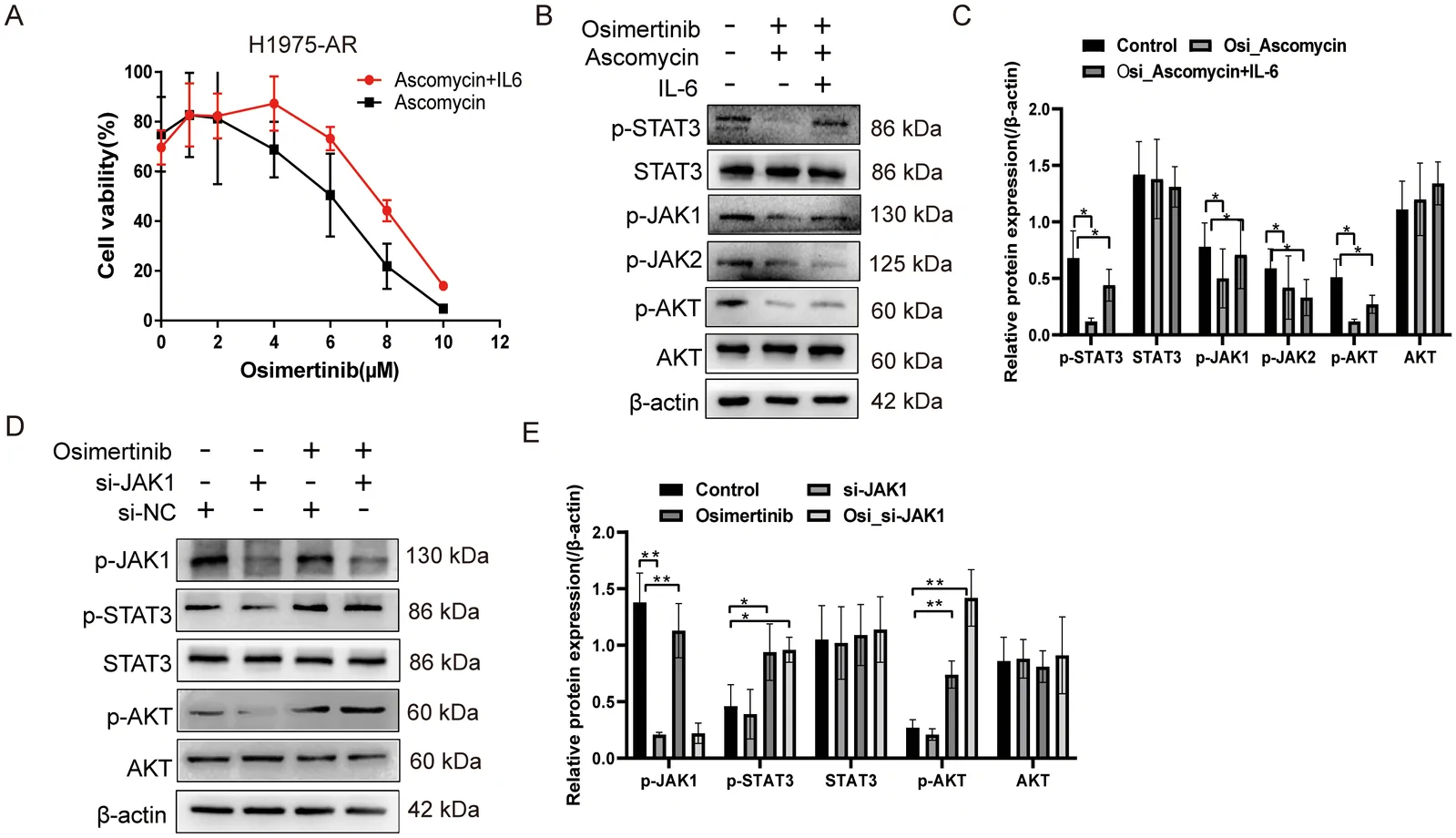
Ascomycin reverses osimertinib resistance through the IL-6/JAK1/STAT3 signaling axis. **(A)** H1975-AR cells were treated with 4 μM ascomycin alone or in combination with IL-6 (10 ng/mL) for 48 h. Cell viability was evaluated using CCK-8 assays. **(B)** H1975-AR cells were treated with 4 μM ascomycin plus 4 μM osimertinib, with or without IL-6 (10 ng/mL), for 48 h. p-JAK1, p-JAK2, STAT3, p-STAT3, AKT, and p-AKT expression was analyzed by western blotting. Representative immunoblots are shown. **(C)** Quantification of protein expression normalized to β-actin. **(D)** H1975-AR cells were transfected with control siRNA or *JAK1* siRNA and treated with or without 4 μM osimertinib for 48 h. p-JAK1, STAT3, p-STAT3, AKT, and p-AKT expression was determined by western blotting. **(E)** Quantification of protein expression normalized to β-actin. Quantitative data are presented as the mean ± SD (n = 3). *p < 0.05, **p < 0.01, one-way ANOVA.

The IL-6/JAK1/STAT3 signaling pathway has critical roles in acquired EGFR-TKI resistance (28). To further establish a causal relationship between ascomycin-mediated IL-6 signaling inhibition and restored osimertinib sensitivity, rescue experiments were performed in H1975-AR cells. Consistent with H1975 cell findings, ascomycin pretreatment significantly increased H1975-AR cell sensitivity to osimertinib; however, this effect was partially reversed by exogenous IL-6 administration (**Figure 5A**). Western blotting showed that ascomycin markedly inhibited STAT3 phosphorylation in H1975-AR cells, whereas concurrent IL-6 exposure treatment partially restored STAT3 activation (**Figures 5B, C**). Previous studies have reported that JAK1 and JAK2 are key mediators of IL-6-induced STAT3 activation (29). Consistent with this, combined ascomycin and osimertinib treatment markedly reduced both JAK1 and JAK2 phosphorylation. Interestingly, IL-6 supplementation significantly increased JAK1 phosphorylation by 42.0% but had little effect on JAK2 phosphorylation, suggesting that STAT3 activation in H1975-AR cells may be primarily dependent on JAK1 signaling (**Figures 5B, C**). To further validate a role for JAK1 in STAT3 activation, JAK1 expression was siRNA-silenced in H1975-AR cells. Compared with the control group, JAK1 knockdown significantly reduced both STAT3 and AKT phosphorylation, confirming a central role for JAK1 in this pathway. Osimertinib treatment also partially reduced p-JAK1, the combination of JAK1 interference and osimertinib further suppressed p-JAK1 levels. Notably, although JAK1 silencing alone reduced phosphorylated STAT3 levels by 57.1% and phosphorylated AKT1 levels by 71.6%, concurrent treatment with JAK1 siRNA and osimertinib resulted in a partial restoration of STAT3 and AKT phosphorylation (**Figures 5D, E**), suggesting that osimertinib-induced STAT3 activation may have partially counteracted JAK1 silencing effects. Thus, ascomycin sensitized osimertinib-resistant H1975-AR cells, at least in part, by suppressing the IL-6/JAK1/STAT3 signaling axis.

### Ascomycin enhances osimertinib antitumor activity *in vivo*

**Figure 6:**
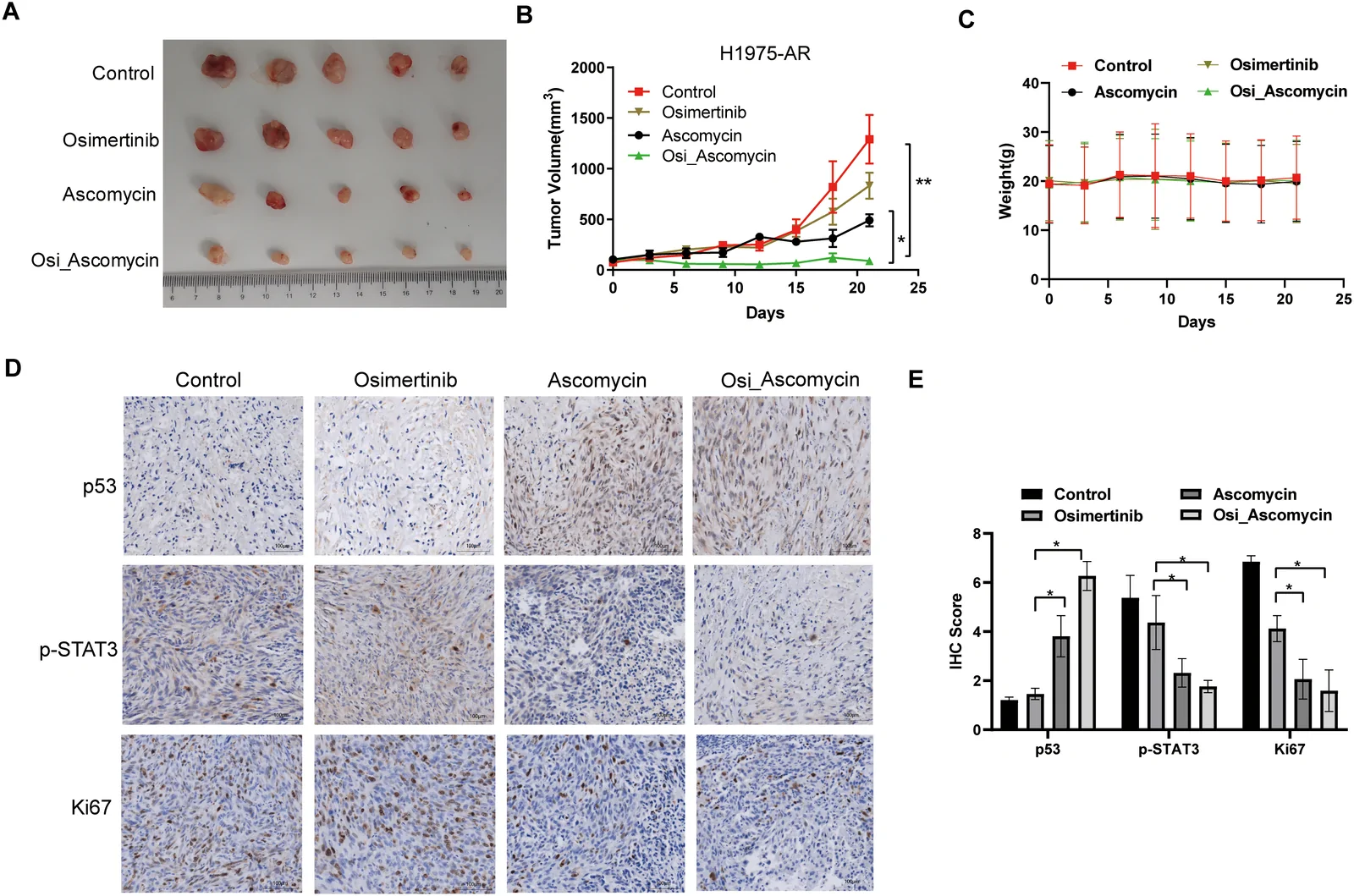
Ascomycin enhances osimertinib antitumor activity *in vivo.* **(A)** Representative images showing xenograft tumors harvested from BALB/c nude mice in vehicle, osimertinib, ascomycin, and combination treatment groups (n = 6/group). **(B, C)** Quantitative analysis of tumor volume and body weight across treatment groups. **(D)** p53, p-STAT3, and Ki-67 expression in xenograft tumor tissues by immunohistochemical (IHC) staining (scale bar = 100 μm). **(E)** Quantitative analysis of IHC staining scores for p53, p-STAT3, and Ki-67. Data are presented as the mean ± SD. Statistical significance was determined by one-way ANOVA.

To evaluate the therapeutic efficacy of combined ascomycin and osimertinib treatment, H1975-AR subcutaneous xenograft models were established in nude mice. Consistent with *in vitro* findings, osimertinib monotherapy inhibited only modest tumor growth in the resistant model compared to the vehicle control group. Ascomycin alone exerted moderate antitumor activity. However, the combined treatment of ascomycin and osimertinib elicited the most potent effect, exhibiting significantly enhanced inhibition compared to either monotherapy (**Figures 6A, B**). Changes in body weight during treatment are shown (**Figure 6C**). No remarkable weight loss was observed in monotherapy groups or the combination group, indicating favorable safety of the combination regimen at the experimental doses.

To further investigate the mechanism underlying this enhanced *in vivo* antitumor efficacy, IHC staining was performed on excised tumor tissues. Immunohistochemical (IHC) analysis revealed osimertinib monotherapy did not significantly alter Ki-67, p-STAT3, or p53 expression levels compared to the vehicle control group (**Figures 6D, E**). Compared with the vehicle control, Ki-67 positivity was significantly reduced by 69.7% in the ascomycin monotherapy group, and by 76.8% in the combination group. Both groups exhibited substantially weaker Ki-67 staining compared with osimertinib monotherapy. Regarding p-STAT3 levels, ascomycin monotherapy resulted in a 56.9% reduction relative to the vehicle control. Combination treatment induced a more pronounced decrease of 79.0%. Notably, p-STAT3 abundance was markedly lower in both the ascomycin monotherapy and combination groups compared to the osimertinib monotherapy group. Conversely, p53 expression was significantly upregulated, showing a 3.15-fold increase in the ascomycin monotherapy group and a 5.18-fold increase in the combination group relative to the vehicle control. The staining intensity of p53 in the combination group was also 4.29-fold higher than in the osimertinib monotherapy group.

## Discussion

Osimertinib has demonstrated substantial clinical efficacy in treating advanced EGFR-mutant NSCLC (30); however, acquired resistance remains a major clinical challenge. Strategies overcoming this resistance, particularly using combination therapies, are essential for improving long-term clinical outcomes (4). In this study, it was demonstrated that ascomycin, a potent CaN phosphatase inhibitor, effectively reversed acquired osimertinib resistance, with a combined ascomycin and osimertinib treatment significantly suppressing tumor growth both *in vitro* and *in vivo.* These findings provide a novel therapeutic strategy for overcoming EGFR-TKI resistance in NSCLC and suggest an approach that could prolong the clinical benefits of targeted therapy.

Ascomycin is a well-characterized immunosuppressive agent that exerts its effects by inhibiting the CaN/NFAT signaling pathway (10, 31). This mechanism suppresses T-cell activation and proliferation, thereby producing potent immunosuppressive activity. Structural ascomycin analogs (e.g., FK506) have demonstrated antitumor activity across several malignancies, including melanoma and bladder cancer (12, 32). Previous studies have also reported that ascomycin suppresses melanoma cell growth, migration, and invasion by inhibiting NFAT3 activation (12). The present study investigated the capacity of ascomycin to overcome osimertinib resistance in NSCLC. Co-treatment with ascomycin and osimertinib significantly potentiated cell growth suppression and apoptosis induction in osimertinib-resistant H1975-AR cells, whereas monotherapy with each individual agent exerted minimal antitumor activity.

Apoptosis is a tightly regulated form of programmed cell death that can be triggered by anticancer therapies, with its induction a well-established strategy used to overcome drug resistance in cancer (33, 34). The present study demonstrated that combined ascomycin and osimertinib treatment significantly induced apoptosis, thereby enhancing osimertinib antitumor efficacy in EGFR-TKI-resistant NSCLC cells both *in vitro* and *in vivo*. Mechanistically, this combination markedly increased expression of the pro-apoptotic proteins Bax and p53 and promoted caspase-3 activation in H1975-AR cells. These findings are consistent with previous reports showing that apoptotic pathway modulation can restore EGFR-TKI sensitivity. For example, pitavastatin sensitizes NSCLC cells to EGFR-TKIs by downregulating BCL-2 and upregulating BAX, thereby shifting the balance toward apoptosis (35). Similarly, the STAT3 inhibitor BBI608 decreases BCL2 expression while increasing p53, BAX, and cleaved caspase-3 levels in gefitinib-resistant HCC827/Ge-r cells, thereby promoting apoptosis and enhancing gefitinib antitumor activity in EGFR-mutant NSCLC (36). Additionally, *YES1* knockdown downregulates anti-apoptotic proteins such as BCL2 and BCL-XL and restores EGFR-TKI sensitivity in HCC827/GR gefitinib-resistant cells (37). Collectively, these findings suggest that targeting survival pathways and apoptotic regulators are promising strategies for overcoming acquired EGFR-TKI resistance in NSCLC.

STAT3 is frequently overexpressed and constitutively activated in several malignancies, where it regulates proliferation, survival, immune evasion, and angiogenesis (38). Increasing evidence now indicates that persistent STAT3 activation is a major mechanism underlying resistance to both conventional chemotherapy and targeted therapies (26). For example, in head and neck squamous cell carcinoma, sustained STAT3 activation is a hallmark of EGFR-TKI resistance, and therapeutic strategies incorporating STAT3 inhibitors have demonstrated significant antitumor activity (39). Zheng et al. showed that the novel STAT3 inhibitor W2014-S restored EGFR-TKI sensitivity in resistant NSCLC models both *in vitro* and *in vivo* (7). Consistent with these observations, markedly elevated STAT3 activation was detected in H1975-AR cells when compared with parental H1975 cells, supporting a close association between osimertinib resistance and STAT3 signaling. Notably, ascomycin treatment significantly reduced STAT3 phosphorylation, while a combination treatment with osimertinib produced more pronounced inhibitory effects than either monotherapy. These findings suggest that ascomycin’s ability to restore osimertinib sensitivity is mediated, at least in part, by suppressing STAT3 signaling. Therefore, these results highlight STAT3 as a promising therapeutic target for patients with NSCLC who develop EGFR-TKI resistance.

IL-6 is a pleiotropic cytokine that promotes tumor progression, immune evasion, and therapeutic resistance in NSCLC (24, 40). The IL-6/JAK/STAT3 signaling cascade is a well-established pro-survival pathway, and its inhibition an attractive strategy for overcoming EGFR-TKI resistance. Previous studies have reported that targeting this pathway could restore sensitivity to TKIs such as afatinib and gefitinib (28, 41). The present findings extend these observations by identifying the IL-6/JAK1/STAT3 signaling axis as a key mediator of acquired osimertinib resistance in H1975-AR cells. Significant pathway activation was observed in resistant cells relative to parental cells. Furthermore, exogenous IL-6 stimulation promoted osimertinib resistance in parental H1975 cells and partially attenuated ascomycin antiproliferative effects in H1975-AR cells. Mechanistically, ascomycin treatment reduced both JAK1 and JAK2 phosphorylation. Interestingly, IL-6 supplementation partially restored JAK1 phosphorylation but had little effect on JAK2 phosphorylation, suggesting that STAT3 activation in resistant cells may be predominantly dependent on JAK1. Although IL-6 secretion, receptor complex formation, and JAK1 kinase activity were not directly examined, the findings establish clear associations between ascomycin treatment and a suppressed IL-6/JAK1/STAT3 signaling pathway. Therefore, these data suggest that ascomycin overcomes acquired osimertinib resistance, at least in part, by inhibiting IL-6/JAK1/STAT3 signaling. As ascomycin is a CaN inhibitor, it is also possible that upstream transcriptional programs involving NFAT-related signaling contribute to these effects; however, this warrants further investigation.

Despite these promising synergistic effects, several study limitations are acknowledged and may inform future research directions. First, although the H1975-AR model provides a robust proof-of-concept system for investigating osimertinib resistance, validation in other resistance models, particularly patient-derived xenograft models, will be required to better define the therapeutic scope and clinical applicability of this combination strategy. Additionally, comprehensive toxicity and survival evaluations are required before clinical translation can be considered. Future preclinical studies should therefore incorporate detailed toxicity assessments, including the histopathological examination of major organs and longitudinal monitoring of body weight, together with Kaplan–Meier survival analyses to evaluate the long-term safety and efficacy of this combination treatment.

A second limitation relates to the pleiotropic biological effects of ascomycin as a CaN inhibitor. Although the experimental design did not permit a comprehensive evaluation of its immunomodulatory activity, CaN signaling regulates multiple biological processes beyond classical immunosuppression, including T-cell activation, cytokine production, and tumor cell-intrinsic signaling pathways. Consequently, the synergistic effects observed here may not be solely attributable to the direct inhibition of tumor cell signaling, but may also involve modulation of the tumor microenvironment. For example, suppression of the IL-6/STAT3 signaling pathway observed here may be partially independent of immune-cell activity, as IL-6 can be produced by both tumor and stromal cells, whereas STAT3 functions as a critical oncogenic regulator in tumor cells.

Several challenges must also be addressed before the ascomycin/osimertinib combination is translated into clinical practice. The systemic immunosuppressive properties of ascomycin may pose potential safety concerns, particularly given increasing immunotherapy roles in lung cancer treatment. Accordingly, future studies should explore the development of ascomycin derivatives that retain EGFR-TKI-sensitizing activity while minimizing immunosuppressive effects. Additionally, as both ascomycin and osimertinib are CYP3A4 substrates, potential pharmacokinetic interactions between these agents require careful investigation. Finally, although the doses used here fell within potentially translatable ranges, comprehensive Good Laboratory Practice-compliant toxicology studies must establish formal preclinical safety profiles before clinical evaluation.

## Conclusions

**Figure 7:**
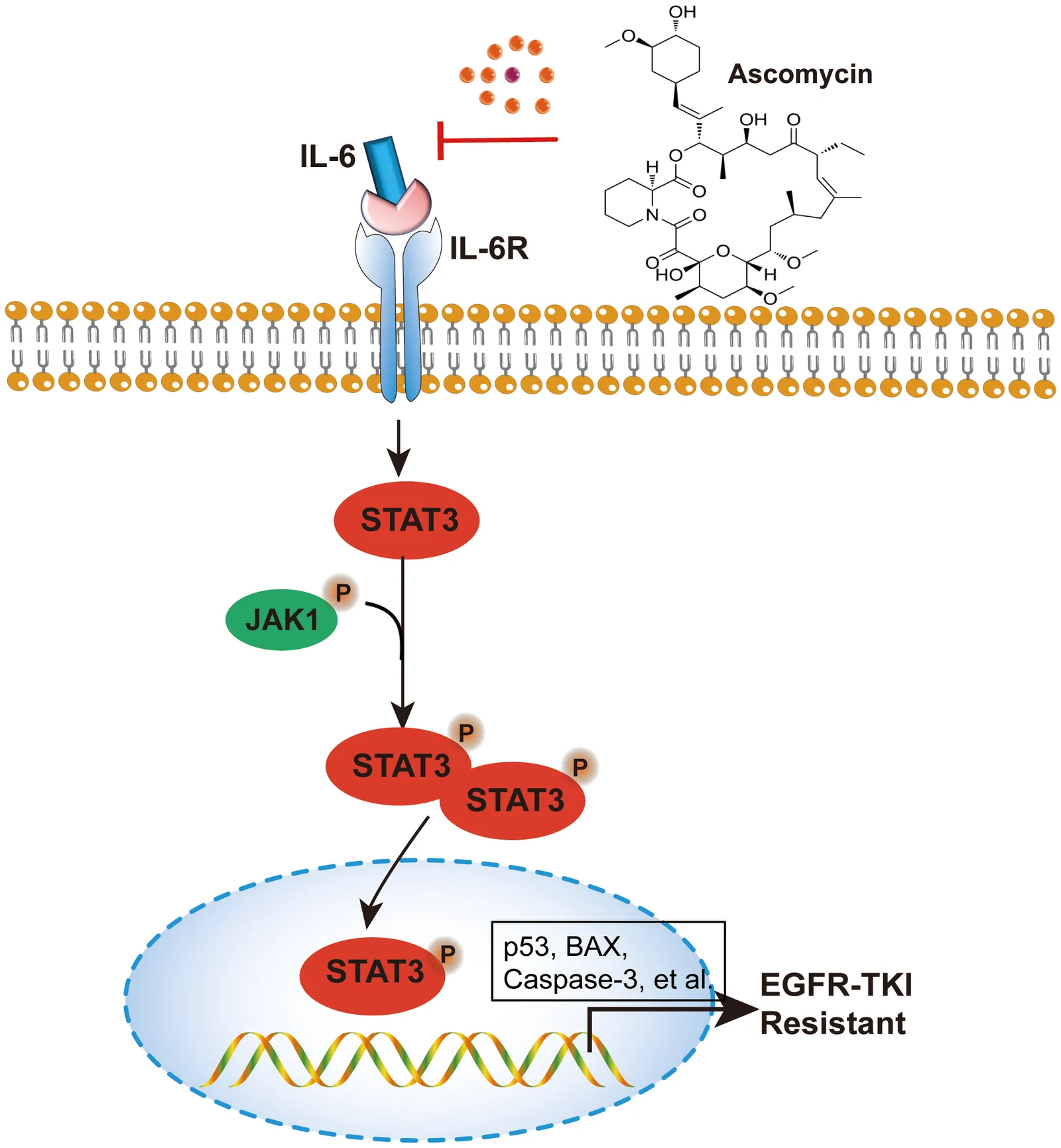
Proposed mechanism whereby ascomycin restores osimertinib sensitivity in osimertinib-resistant lung cancer cells.

Collective findings demonstrate that ascomycin enhances the antiproliferative activity of osimertinib in EGFR-TKI-resistant NSCLC cells. The inhibitory regulation of the IL-6/JAK1/STAT3 signaling cascade is presumed to be partially responsible for the above therapeutic effect (**Figure 7**). Combined therapy consisting of ascomycin and osimertinib exerts prominent inhibitory effects on tumor growth in both *in vitro* and *in vivo* acquired osimertinib resistance models. These observations highlight combination of EGFR-TKIs with ascomycin, or other agents targeting STAT3 signaling, as a promising therapeutic strategy to reverse acquired resistance and optimize clinical treatment outcomes in patients with advanced NSCLC.

## Data Availability

The datasets used and/or analyzed during the current study are available from the corresponding author on reasonable request.
